# Controlling for confounding factors and revealing their interactions in genetic association meta-analyses: a computing method and application for stratification analyses

**DOI:** 10.18632/oncotarget.24335

**Published:** 2018-01-29

**Authors:** Shuhuang Lin, Xu Liu, Bin Yao, Zunnan Huang

**Affiliations:** ^1^ Key Laboratory for Medical Molecular Diagnostics of Guangdong Province, Dongguan Scientific Research Center, Guangdong Medical University, Dongguan, Guangdong 523808, China; ^2^ The Second School of Clinical Medicine, Guangdong Medical University, Dongguan, Guangdong 523808, China; ^3^ Institute of Marine Biomedical Research, Guangdong Medical University, Zhanjiang, Guangdong 524023, China

**Keywords:** meta-analysis, stratification analysis, subgroup analysis, confounding control, interacting effect

## Abstract

Subgroup and stratification analyses have been widely applied in genetic association studies to compare the effects of different factors or control for the effects of the confounding variables associated with a disease. However, studies have not systematically provided application standards and computing methods for stratification analyses. Based on the Mantel-Haenszel and Inverse-Variant approaches and two practical computing methods described in previous studies, we propose a standard stratification method for meta-analyses that contains two sequential steps: factorial stratification analysis and confounder-controlling stratification analysis. Examples of genetic association meta-analyses are used to illustrate these points. The standard stratification analysis method identifies interacting effects on investigated factors and controls for confounding variables, and this method effectively reveals the real effects of these factors and confounding variables on a disease in an overall study population. We also discuss important issues concerning stratification for meta-analyses, such as conceptual confusion between subgroup and stratification analyses, and incorrect calculations previously used for factorial stratification analyses. This standard stratification method will have extensive applications in future research for increasing studies on the complicated relationships between genetics and disease.

## INTRODUCTION

Meta-analysis is a powerful and effective tool in evidence-based medicine (EBM) for synthesizing results from multiple case-controlled studies into a single numerical estimate to provide more objective and quantitative evidence. This method has been widely applied over the past two decades to solve clinical problems in numerous genetic association studies [1–4]. Meta-analyses are convenient for large-scale, multi-centre and multivariate investigations between genetic polymorphisms and disease development. In meta-analyses, subgroup analysis and stratification analysis are frequently used to compare the sizes of the effects of variants of an intervention or to control for confounding factors to clarify the real effects of the intervention. These two methods are quite different methodologically.

### Subgroup analysis

In the meta-analysis approach, subgroups are based on a characteristic of the included studies. In addition to exploring the source of heterogeneity between studies, subgroup analyses are conducted to determine whether differences occur among studies with two or more different levels of a characteristic, and the characteristics are then compared. In this method, a study is considered a unit, and studies with the same features are considered a subset. For example, such analyses may be performed to determine whether a genetic polymorphism has different effects among different ethnicities. Then, all the included studies are divided into several groups, such as Asian, Caucasian and African. Odds ratios (ORs) with confidence intervals (CIs) are calculated within the different groups and then compared across the groups. The study by Borenstein M discusses the detailed application standards, computing methods and results interpretation of this method [5].

### Stratification analysis

The term “stratification” means that the study population is divided into several strata according to characteristics that may influence the clinical indexes. Stratified data are generally sorted as in Table 1. In a case-controlled study, stratification analysis (also called stratified analysis [6] or risk stratification analysis [7] when the confounding factor is a risk factor for the disease) is a method of controlling the confounding factors and revealing the true relationship between exposure and disease by calculating the sizes of the effects within the stratum, with a Mantel-Haenszel approach used to combine them. This method is also called M-H stratified analysis, and its application in a single case-controlled study is defined and described thoroughly by Mantel N [6] and Hill A [8].

**Table 1:** Sorting table for stratified data in a case-controlled study

Exposure	Stratum i		Total
	Cases	Controls	
+	a_i_	b_i_	n_1i_
–	c_i_	d_i_	n_0i_
Total	m_1i_	m_0i_	t_i_

**Figure 1:**
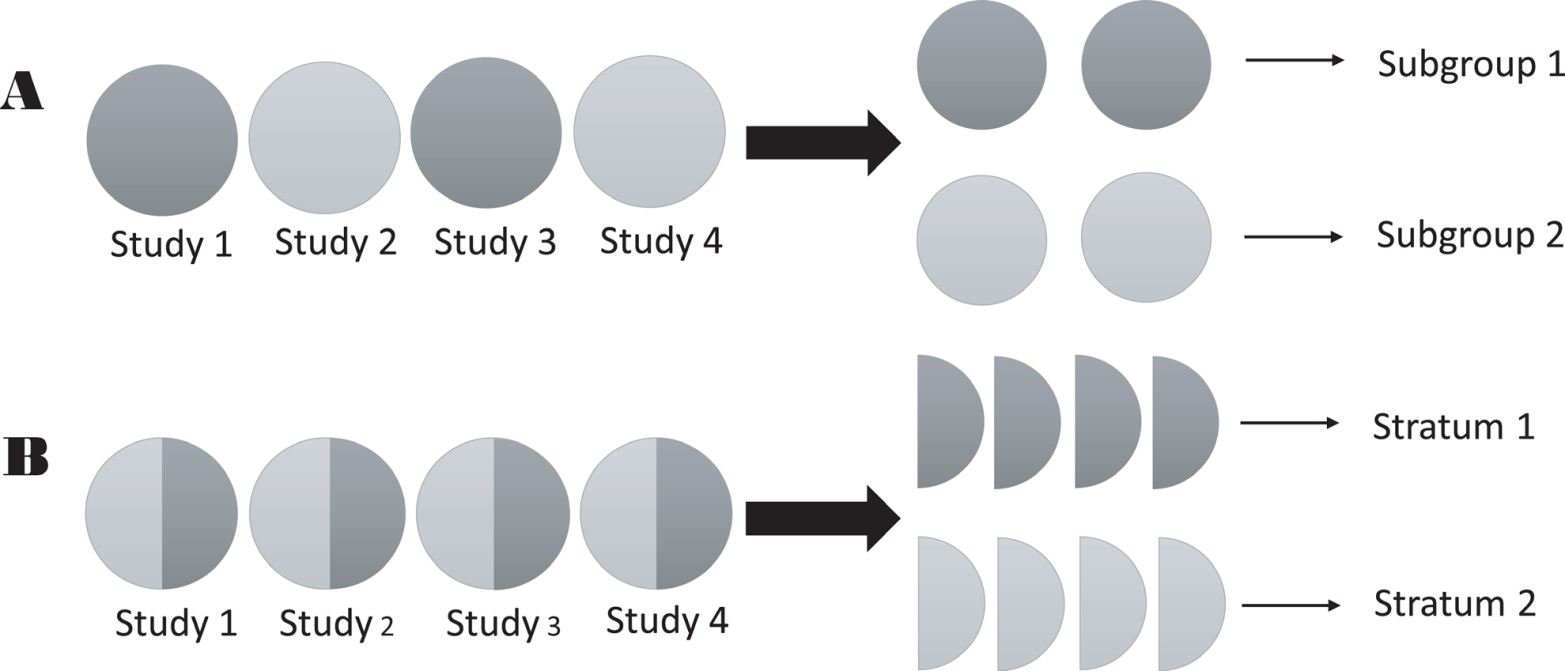
Schematic of the difference between strata and subgroups in meta-analyses (**A**) subgroup analysis; and (**B**) stratification analysis.

As above, stratification is different from subgroups in two aspects: (a) in a single study, grouped indicators are generally interventions that must be pre-set, whereas stratified indicators are often considered potential confounding variables; (b) in a meta-analysis, an included study is considered a unit and assigned to one group in a subgroup analysis, whereas in a stratification analysis, an included study is divided into several parts, and studies with identical characteristics will form the same strata. This difference is shown in a schematic (Figure 1). One of the purposes of distinguishing subgroup and stratification analyses is to emphasize that only stratified data can be used for stratification analysis in a meta-analysis.

Two methods of calculating the ORs for stratified data are found in published meta-analyses: (a) with the first method, the unexposed group in a stratum is regarded as a reference and compared with the exposed group in the same stratum, the unexposed group in another stratum and the exposed group in the other stratum [9–11]; (b) with the second method, the ORs are calculated within the same stratum and then compared [12–14]. This difference can be easily understood visually (Table 2).

**Table 2:** Two practical variants of stratified analysis in previous genetic association meta-analyses

(A)				
Exposure	Stratum 1	Stratum 2	…	Stratum i
–	Reference	OR_2–_	…	OR_i–_
+	OR_1+_	OR_2+_	…	OR_i+_
**(B)**				
**Exposure**	**Stratum 1**	**Stratum 2**	**…**	**Stratum i**
–	Reference	Reference	…	Reference
+	OR_1_	OR_2_	…	OR_i_

Although stratification analyses have been widely used by many investigators to study the association of genes with disease in meta-analyses [9–15], studies have not previously provided a systematic description of the application standards, computing methods and results interpretation, which we intend to classify in this work. Referring to the M-H stratified analysis used in single case-controlled studies and the two practical methods of calculating ORs mentioned above, this study provides a standard method of performing stratification analyses for use in meta-analyses. To illustrate this point in detail, we use three examples of meta-analyses related to genetic associations in the development of cancer or Alzheimer’s disease (AD).

### Examples for illustration

The first study used here for illustrative purposes was conducted by Nagao M *et al.* [13], who investigated the roles of a prostaglandin endoperoxide synthase (*PTGS2*) polymorphism and nonsteroidal anti-inflammatory drug (NSAID) intake in the risk of developing cancer. In the second and third meta-analyses, we focused on the studies of Li L *et al.* [14] and Zhang MY *et al.* [15], who investigated the associations of cholesterol-24S-hydroxylase (*CYP46A1*) rs754203 and methylenetetrahydrofolate reductase (*MTHFR*) rs1801133 polymorphisms with AD according to apolipoprotein E ε4 (*ApoE* ε4) status. The original data included in these three meta-analyses are displayed in Supplementary Table 1.

In this paper, we focus on the methodological aspects of statistical analyses rather than other issues, such as the source of the data and quality assessments of the studies. We will not discuss these substantive issues. Readers who are interested in the details of these three meta-analyses can refer to the original papers.

#### PTGS2 polymorphism and NSAID use in the risk of cancer

This meta-analysis included eight case-controlled studies containing a total of 3032 cancer patients and 5712 controls [13], and all include stratified data. The study population can be divided by *PTGS2* rs5275 genotype carrier status (TC or CC carriers and TT carriers) or stratified by NSAID use history (NSAID users and non-NSAID users), as shown in Supplementary Table 1. In this meta-analysis, the researchers had two goals: (a) to determine the relationship between the *PTGS2* rs5275 polymorphism and cancer risk and (b) to investigate the effect of NSAID use on preventing cancer.

#### CYP46A1 or MTHFR polymorphisms by ApoE ε4 status in the risk of AD

These two meta-analyses included eight and six case-controlled studies containing a total of 1700 AD patients and 1433 controls [14] and 1063 AD patients and 1151 controls [15], respectively. Among all the study subjects, *CYP46A1* rs754203 (TT, TC or CC carriers, T was the major allele) or *MTHFR* rs1801133 (CC, CT or TT carriers, C was the major allele) polymorphisms and ApoEε4 carrier status (non-ApoE ε4 carriers and ApoE ε4 carriers) were determined by genotyping. The stratified data are shown in Supplementary Table 1. The researchers of these two meta-analyses investigated (a) the associations of *CYP46A1* rs754203 or *MTHFR* rs1801133 polymorphisms with the risk of developing AD and (b) the confounding or interaction effect of ApoE ε4.

It would be difficult for a simple meta-analysis to fully utilize the stratified data of the case-controlled studies above to interpret the complicated relationship between gene-gene or gene-environment effects and disease risk. In the following sections, we will introduce the computing method and discuss the application of a standard stratification analysis to solve these issues in meta-analyses.

### Methodology of stratification analysis in meta-analyses

For all selected examples in our paper, ORs with corresponding 95% CIs were selected as the effect sizes to assess the associations between the single nucleotide polymorphisms (SNPs) and the risks of the diseases. An OR with a 95% CI greater than 1 indicates that the investigated factor increased the risk of the disease, and an OR with a 95% CI less than 1 indicates that the investigated factor decreased the risk of the disease. When an OR with a 95% CI crosses 1, no significant association is indicated between the investigated factor and the disease.

**Figure 2:**
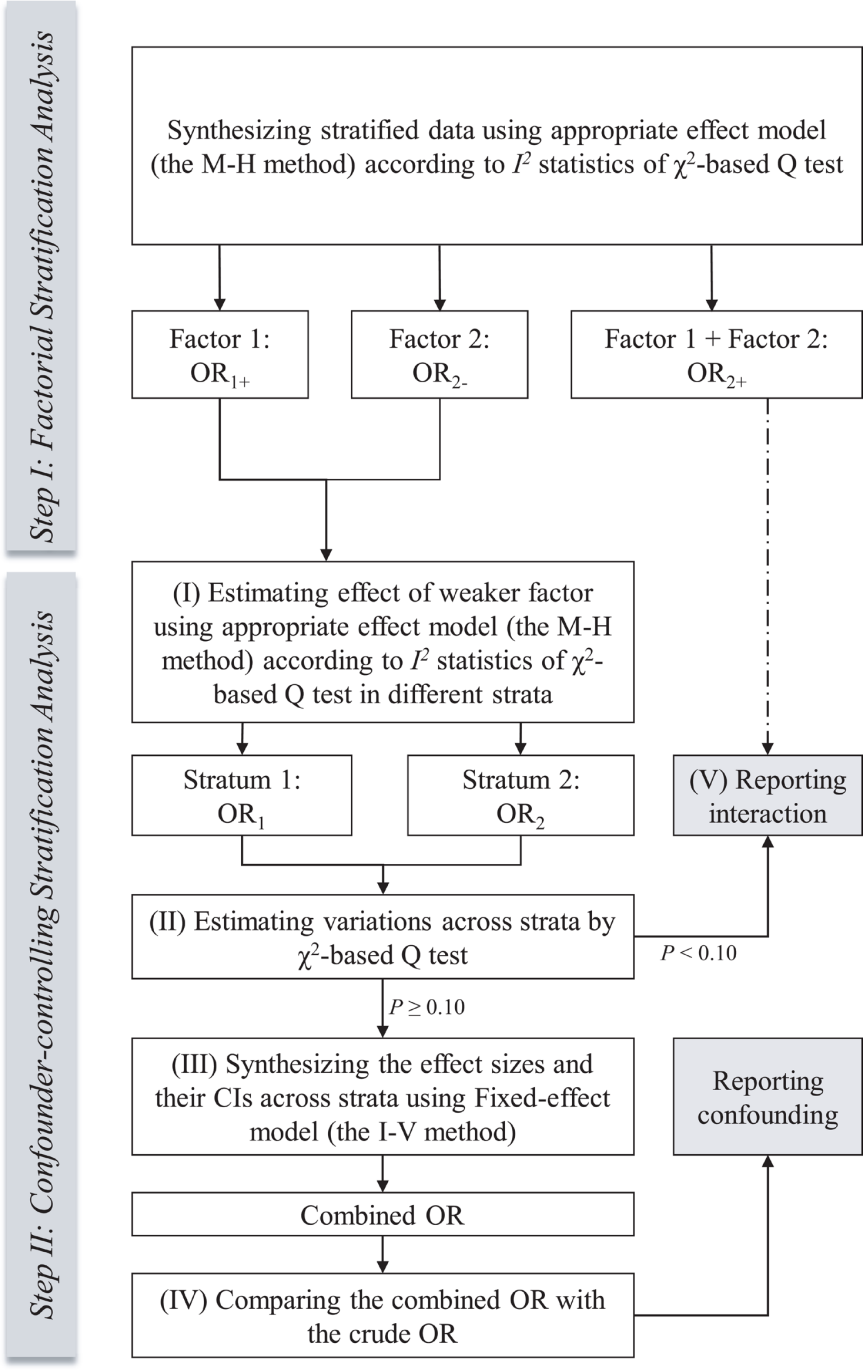
Flow diagram of the process of standard stratification analysis in meta-analysis

In each meta-analysis, the ORs were calculated for stratified data in two sequential steps for a standard stratification analysis (Figure 2):

- In the first step, the computing method for ORs refers to the first type of variants of stratification analysis (Table 2A) found in previous published meta-analyses. These ORs reveal the effect of the stratifying indicator within the unexposed group (OR_2-_), the effect of the exposure factor within the same stratum (OR_1+_) and the effect of both the stratifying indicator and exposure factor (OR_2+_). We call this “factorial stratification analysis”. Appropriate effect models are selected to calculate the pooled ORs based on the heterogeneity test. Heterogeneity is evaluated according to the χ^2^-based Q (Cochran’s *Q* test) and *I*^*2*^ statistic tests [16]. If *I*^*2*^ < 50%, then the fixed-effect model (the Mantel-Haenszel method) [6] is applied. Otherwise, we select the random-effects model (the Der Simonian-Laird method) [17]. This model selection criterion has long been used in meta-analyses.
- In the second step, the computing method for ORs refers to the second type of variants of stratification analysis (Table 2B) found in previous published meta-analyses. The weaker effect between two factors mentioned above is investigated first, and then the stronger one is investigated (OR_1+_ vs. OR_2-_). The true role of the weaker factor is more difficult to detect under the confounding influence of the stronger one. Figure 2 describes the following sub-steps to control for the confounding influence of the stratified moderator effect. We call this “confounder-controlling stratification analysis”.

(I) When working within strata, the process of effect model selection is the same as that of normal meta-analysis and factorial stratification analysis mentioned above. Proper effect models are used to pool the stratified data of every included study within each stratum. Because this sub-step is similar to the subgroup analysis, we call this “subgroup-type stratification analysis”.

- Traditional subgroup-typed stratification analysis without combining the sizes of effects across strata can show only the investigated effect in a particular subpopulation, which may not reveal the real effect among all study populations. In addition, although observed effects may be different across strata due to the confounding factors, their true values may be consistent. To solve this problem, we use the test of homogeneity across strata to determine whether to combine the effects of different strata. The variation across strata is then assessed. The χ^2^- based *Q* test is also conducted for this evaluation.
- If the *P* value of the χ^2^ statistic ≥0.1, no significant variation is indicated as shown in the test of homogeneity. An overall estimation is determined for the effect of the investigated factor. The fixed-effect model (the Inverse-Variance method) is used to combine the effect size with the upper and lower CIs of each strata because the fixed-effect model assumes a common true effect size (fixed-effect) or different but countable true effect sizes (fixed-effects) across strata [5, 6, 18].
- Then, the adjusted OR by stratification, or the combined OR, is compared with the crude OR calculated from the overall study populations in a simple meta-analysis without stratification. Any change in significance between these values indicates that the stratified moderator is a confounding variable.
- If the *P* value of the χ^2^ statistic <0.1, significant variation across strata is indicated. In this case, we do not use a random-effects model to combine the ORs from different strata. The random-effects model assumes that true effect sizes are distributed across strata and that the number of strata is countless [5, 17, 18]. So, using a random-effect model to combine these ORs is inappropriate. Therefore, we do not combine these ORs, but we use them and OR_2+_ to report the unilateral effect of the investigated factor in each stratum as well as the interacting effect between this factor and the stratified moderator on diseases, respectively.

A factorial stratification analysis can be used to determine whether exposure or confounding factors have a multilevel perspective, especially the simple effect of a confounding factor in a study population, which cannot be provided by the subgroup-typed calculation method. In confounder-controlling stratification analysis, we can control for the confounding variable via stratification, determine the real effects of exposure factors, or discover whether interactions occur between exposure and confounding factors. This interaction is referred to as a gene-gene or gene-environment interaction. In a standard stratification analysis, these two sub-types of stratification methods are included as the first step and the second step.

All analyses were performed using STATA version 14.0 software (STATA Corporation, College Station, TX, USA) with the *metan* command [19]. The above algorithms of Cochran’s *Q* test and the fixed-effect and random-effects models refer to the *Cochrane Handbook for Systematic Reviews of Interventions: Version 5.1.0* [20] and the method of genetic association meta-analysis by Thakkinstian M *et al.* [21]. The code of command line in Stata is displayed in the Supplementary Material.

### Application and results interpretation

#### PTGS2 polymorphism and NSAID use in the risk of cancer

In the original publication, the researchers calculated four pooled ORs using a subgroup-type stratification analysis: (a) the minor allele carriers compared with the carriers of the homozygous major allele *PTGS2* rs5275 among NSAID users (TC + CC vs. TT: OR = 1.008, *P* = 0.916); (b) the minor allele carriers compared with the carriers of the homozygous major allele *PTGS2* rs5275 among non-NSAID users (TC + CC vs. TT: OR = 0.934, *P* = 0.264); (c) the NSAID users compared with the non-NSAID users among the homozygous major allele carriers of *PTGS2* rs5275 (NSAID users vs. non-NSAID users: OR = 0.841, *P* = 0.01); and (d) the NSAID users compared with the non-NSAID users among the minor allele carriers of *PTGS2* rs5275 (NSAID users vs. non-NSAID users: OR = 0.769, *P* < 0.001). The data are shown in Supplementary Table 2. Based on these statistical results, they concluded that NSAID use decreased the risk of cancer among both TC or CC carriers and TT carriers, whereas the *PTGS2* rs5275 polymorphism did not show any significant effect on cancer development among NSAID users or non-NSAID users [13]. Although confounding factors were controlled within a stratum, these results were limited due to the two following issues: (a) the effect of either the *PTGS2* rs5275 polymorphism or NSAID use in the overall study population was not explored in this study; and (b) repeated pairwise comparisons increased the potential rate of type I errors in the meta-analysis, which may lead to false-positive results.

In our work, we performed a standard stratification analysis to investigate the relationship of the *PTGS2* polymorphism and NSAID use with the risk of cancer following the steps below. First, since the effects of both the *PTGS2* polymorphism and NSAID use were unclear before our analysis, a factorial stratification analysis was used to observe the effect sizes. As shown in Table 3, the unilateral effect of the *PTGS2* rs5275 polymorphism did not significantly change the risk of developing cancer (OR = 0.934, *P* = 0.264), although the unilateral effect of NSAID use decreased this risk significantly (OR = 0.769, *P* < 0.001), supporting the conclusion in the study by Nago M *et al.* [13]. When both of the above effects occurred, the ORs did not show a significant difference from that of the unilateral effect of NSAID use (OR = 0.76, *P* < 0.001). These ORs implied that only NSAID use may have a protective role in cancer development and the *PTGS2* rs5275 polymorphism was not significantly associated with this risk.

**Table 3:** Meta-analysis stratified by NSAID use status to determine the association between the PTGS2 rs5275 polymorphism and the risk of cancer

Gene local	No. of studies	Steps for standard stratification analysis	Genetic comparison	Non-NSAID user					NSAID user				
				*I*^*2*^	Model	OR	95% CI	*P*	*I*^*2*^	Model	OR	95% CI	*P*
*PTGS2*	8	**I**	**TT**	NA		1 (reference)		NA	48.60%	F	0.769	0.665–0.889	<0.001
rs5275			**TC + CC**	0.00%	F	0.934	0.828–1.053	0.264	45.70%	F	0.76	0.662–0.873	<0.001
		**II**	**TT**	NA		1 (reference)		NA	NA		1 (reference)		NA
			**TC + CC**	0.00%	F	0.934	0.828–1.053	0.264	49.80%	F	1.008	0.872–1.165	0.916
**Combined Result: χ2 = 0.62, *P* = 0.432, OR (95% CI) = 0.961 (0.872–1.051)**													

Note: OR: odds ratio; CI: confidence interval; F: fixed-effect model; NA: not available; I: factorial stratification analysis; II: confounder-controlling stratification analysis; PTGS2: prostaglandin endoperoxide synthase 2; NSAID: nonsteroidal anti-inflammatory drug.

As shown by the earlier analysis, the effect of the *PTGS2* rs5275 polymorphism was much weaker than that of NSAID use. We chose to study the effect of the *PTGS2* rs5275 polymorphism. In the following analysis of the effect of the *PTGS2* rs5275 polymorphism in the overall study population rather than that of NSAID use status, it was necessary to control for the potential confounding effect of NSAID use. As shown in Table 3, in a confounder-controlling stratification analysis, the χ^2^-based *Q* test showed no significant difference across strata (χ^2^ = 0.62, *P* = 0.432). The combined OR demonstrated that the *PTGS2* rs5275 polymorphism was not associated with cancer risk in the overall study population (OR = 0.961, 95% CI = 0.872–1.051). Similarly, to investigate the effect of NSAID use on the risk of cancer, we can calculate the crude pooled OR (OR = 0.807, 95% CI = 0.732–0.890, *P* < 0.001, not shown in the tables) because the *PTGS2* rs5275 polymorphism did not have an influence on this risk. In summary, in the standard stratification analysis, (a) the real effects of both the *PTGS2* rs5275 polymorphism and NSAID use on cancer risk were determined in the overall population after controlling for confounding variables, and (b) a standard computing process could mitigate increases in type I errors that may be caused by repeated pairwise comparisons.

### The CYP46A1 and MTHFR polymorphisms in the risk of AD according to ApoE ε4 status

Many genetic association analyses demonstrated that ApoE ε4 represents the most important genetic risk factor for AD [22]. Therefore, when Li L *et al.* [14] and Zhang MY *et al.* [15] investigated the association between the *CYP46A1* and *MTHFR* polymorphisms and AD, the effect of the ApoE ε4 allele was considered because different ApoE ε4 allele distributions between exposed and unexposed groups may represent a confounding variable.

In the second example, researchers calculated the pooled ORs of homozygous mutant genotype CC compared with the TC or TT genotypes of *CYP46A1* rs754203 among ApoE ε4 carriers and non-ApoE ε4 carriers [14]. In the third example, researchers conducted a meta-analysis to investigate the association between the *MTHFR* rs1801133 polymorphism and the risk of AD according to ApoE ε4 status [15]. However, they calculated only the pooled OR among non-ApoE ε4 carriers to exclude the influence of this risk allele on the disease. In contrast, we complemented the pooled OR in ApoE ε4 carriers. The data are shown in Supplementary Table 3. Both the *CYP46A1* rs754203 (OR = 1.528, *P* = 0.018) and *MTHFR* rs1801133 (OR = 1.557, *P* = 0.009) polymorphisms showed significant associations with the risk of AD among non-ApoE ε4 carriers, but not among ApoE ε4 carriers (*P* = 0.284 and *P* = 0.086). However, the results of these two meta-analyses were still limited due to the following issues: (a) in the study of the *CYP46A1* rs754203 polymorphism, the researchers assumed that the negative results in the ApoE ε4 carrier group were caused by stronger effects of the ApoE ε4 allele compared to those of the above polymorphisms [14], although they failed to quantitatively demonstrate this conclusion; and (b) in the study of the *MTHFR* rs1801133 polymorphism, the researchers performed a meta-analysis of non-ApoE ε4 carriers but not ApoE ε4 carriers [15]; thus, an unconventionally negative result, which could easily occur, would be difficult to explain. Different effects of these two polymorphisms observed in different populations with and without ApoE ε4 alleles implied that ApoE ε4 carrier status was a possible confounding factor; however, these studies failed to control for this confounding and reveal the real effects of these two polymorphisms in the overall population.

In our study, a standard stratification analysis was conducted to solve the above issues in these two meta-analyses. For the *CYP46A1* rs754203 polymorphism, the statistical results of the factorial stratification analysis are shown in Table 4. As the data show, the *CYP46A1* rs754203 polymorphism was significantly associated with AD among the non-ApoE ε4 carriers (OR = 1.528, P = 0.018). Additionally, positive ApoE ε4 status increased the risk of developing AD (OR = 5.184, *P* < 0.001), and the OR was 3.39-times greater than that of the *CYP46A1* rs754203 polymorphism. With the combined effect of the *CYP46A1* rs754203 polymorphism and positive ApoE ε4 status, the OR increased compared with that for ApoE ε4 only, indicating a significantly increased AD risk (OR = 7.725, *P* < 0.001). For the *MTHFR* rs1801133 polymorphism, data from the factorial stratification analysis are also shown in Table 4. The statistical results show that the *MTHFR* rs1801133 polymorphism significantly increased the AD risk (OR = 1.557, *P* = 0.009) and also verified that ApoE ε4 was a significant risk factor for AD (OR = 3.678, *P* = 0.001). The OR for the effect of ApoE ε4 on the increasing the AD risk was 2.36-times greater than that for the effect of the *MTHFR* rs1801133 polymorphism. With the combined effect of the *MTHFR* rs1801133 polymorphism and positive ApoE ε4 status, the OR increased from that for the effect of ApoE ε4 alone, indicating a significantly increased AD risk (OR = 6.29, *P* < 0.001). Compared with the subgroup-typed stratification analysis conducted in the original meta-analyses, the factorial stratification analysis provided insight into the role of ApoE ε4 and its combined effect with the *CYP46A1* rs754203 or *MTHFR* rs1801133 polymorphism on the risk of AD.

**Table 4:** Meta-analysis stratified by ApoE ε4 status to determine the association between the CYP46A1 rs754203 or MTHFR rs1801133 polymorphism and the risk of Alzheimer’s disease

Gene local	No. of studies	Steps for standard stratification analysis	Genetic comparison	Non-ApoE ε4 carrier					ApoE ε4 carrier				
				*I*^*2*^	Model	OR	95% CI	*P*	*I*^*2*^	Model	OR	95% CI	*P*
*CYP46A1*	8	**I**	**TT + TC**	NA		1 (reference)		NA	50.80%	R	5.184	3.980–6.753	<0.001
rs754203			**CC**	0.00%	F	1.528	1.075–2.172	0.018	23.90%	F	7.725	4.598–12.978	<0.001
		**II**	**TT + TC**	NA		1 (reference)		NA	NA		1 (reference)		NA
			**CC**	0.00%	F	1.528	1.075–2.172	0.018	33.50%	F	1.33	0.790–2.239	0.284
**Combined Result: χ2 = 0.18, *P* = 0.669, OR (95% CI) = 1.456 (1.019 - 1.893)**													
*MTHFR*	6	**I**	**CC**	NA		1 (reference)		NA	75.60%	R	3.678	1.733–7.806	0.001
rs1801133			**TT**	39.50%	F	1.557	1.119–2.165	0.009	61.50%	R	6.29	2.448–16.159	<0.001
		**II**	**CC**	NA		1 (reference)		NA	NA		1 (reference)		NA
			**TT**	39.50%	F	1.557	1.119–2.165	0.009	12.70%	F	1.619	0.935–2.805	0.086
**Combined Result: χ2 = 0.01, *P* = 0.910, OR (95% CI) = 1.572 (1.115–2.028)**													

Note: OR: odds ratio; CI: confidence interval; F: fixed-effect model; R: random-effect model; NA: not available; I: factorial stratification analysis; II: confounder-controlling stratification analysis; CYP46A1: cholesterol-24 S-hydroxylase; ApoE ε4: the ε4 allele of the apolipoprotein E gene; MTHFR: methylenetetrahydrofolate reductase.

As shown in the earlier analysis, the effect of the *CYP46A1* rs754203 or *MTHFR* rs1801133 polymorphism was much weaker than that of ApoE ε4. We chose to study the effects of the *CYP46A1* rs754203 and *MTHFR* rs1801133 polymorphisms and control for ApoE ε4 as a potential confounding variable in the analyses. We examined the variation between the two ORs calculated in the following confounder-controlling stratification analysis in each polymorphism study based on the χ^2^-based *Q* test. Significant variations were not observed between the results (χ^2^ = 0.18, *P* = 0.669 and χ^2^ = 0.01, *P* = 0.910); therefore, the fixed-effect model was used to combine these ORs (shown in Table 4). In the study of the *CYP46A1* rs754203 polymorphism, the combined OR with a 95% CI was 1.456 (1.019–1.893), which is slightly more pronounced than the crude OR (OR = 1.20, 95% CI = 1.040–1.380) [14]. Both results show that the *CYP46A1* rs754203 polymorphism significantly increased the AD risk in the overall study population. In the study of the *MTHFR* rs1801133 polymorphism, the combined OR with a 95% CI was 1.572 (1.115–2.028), indicating that the *MTHFR* rs1801133 polymorphism also increased the AD risk in the overall study population significantly. However, the crude OR, which was not calculated in the original paper [15] but was complemented by our calculation, did not show that the association was statistically significant (OR = 1.475, 95% CI = 0.962–2.263, *P* = 0.069, not shown in the tables). Therefore, the ApoE ε4 allele had a strong confounding effect on the association. Consequently, it would be difficult to show the real effect of the *MTHFR* rs1801133 polymorphism on the AD risk in the overall study population rather than among the non-ApoE ε4 carriers only without confounder-controlling stratification.

## DISCUSSION

In this study, we focus on providing a systematic study of stratification analyses. Based on the M-H and I-V approaches [6] as well as two practical computational methods used in previous stratification analyses [10, 13, 14], we propose a standard stratification analysis application for meta-analyses that contains two sequential steps: factorial stratification analysis (*Step I*) and confounder-controlling stratification analysis (*Step II*). Three examples of previous meta-analyses meeting the criterion of stratified data are used for illustration.

In the first example, because of the unknown effects of both the *PTGS2* rs5275 polymorphism and NSAID use on the risk of cancer, we calculated ORs using the factorial stratification analysis, which is an earlier stratified method of exploring the unilateral and additive effects of genetic and pharmaceutical factors. A confounder-controlling stratification analysis was further employed to control for the confounding effects of NSAID use during the analysis of the effects of the *PTGS2* rs5275 polymorphism. The combined OR across stratum showed that the *PTGS2* rs5275 polymorphism was not associated with the risk of cancer in the overall study population. To analyse the effect of NSAID use, we directly calculated the crude OR by the fixed-effect model without stratification because the *PTGS2* rs5275 polymorphism was found to have no influence on the risk of cancer.

In the second and third examples, ApoE ε4 was defined as a risk factor for AD [22]. During the investigation of the association of the *CYP46A1* rs754203 and *MTHFR* rs1801133 polymorphisms with the risk of AD, a factorial stratification analysis was first used to verify the role of ApoE ε4 in the disease, and then the second step was carried out to control for the confounding factors. In the study of the *CYP46A1* rs754203 polymorphism, we found that the adjusted OR by stratification was slightly higher than the crude OR, although both were larger than 1 with *P* values smaller than 0.05, suggesting that the *CYP46A1* rs754203 polymorphism was significantly associated with the increased AD risk in the overall population. In the study of the *MTHFR* rs1801133 polymorphism, the adjusted OR by stratification suggested that this polymorphism increased the risk of AD significantly, but it showed no effect on the AD risk by the crude OR. We consider our adjusted OR to be more reliable than the crude OR, which explains the real relationship between the *MTHFR* rs1801133 polymorphism and the AD risk. This finding is reasonable because combining the effect sizes based on the heterogeneity in each stratum first and then across strata provides better results than using the overall heterogeneity among studies in the meta-analysis. The difference between these two ORs implied that ApoE ε4 status was a strong confounder in this meta-analysis. Compared with the results of the unilateral analysis for the *MTHFR* rs1801133 polymorphism in individuals not carrying ApoE ε4 alleles in the original meta-analysis, the result from using both the ApoE ε4 and non-ApoE ε4 carriers in our standard stratification analysis had stronger statistical power because of the larger sample size. The cases mentioned above demonstrate the important role of standard stratification analysis in the exploration and control of confounding factors in meta-analyses.

### Nomenclature

In the Introduction, the difference between the subgroup and stratification concepts was comprehensively discussed. One point that we should stress is that conceptual confusion between the subgroup and stratification concepts occurs in meta-analyses. For example, in a study of the association between the *LIPC* rs493258 polymorphism and the risk of macular degeneration [23], the terms “stratified analysis” and “stratification” were used to describe a subgroup method; however, in the original publication of the study on the relationship of the *VEGF* rs157036 polymorphism with AD risk, a subgroup analysis of ApoE ε4 status was mistaken for a stratified analysis [12]. In the publication used in the first case above, the terms “subgroup analysis” and “stratified analysis” were used to describe the grouping by ethnicity and the types of cancers, respectively [13]. As mentioned earlier, one of the distinctions between strata and subgroups in meta-analyses is that the number of studies will be reduced within groups after grouping but not within strata after stratifying. We know that the number of studies influences the results of the heterogeneity assessment. Therefore, these two methods must be distinguished, which was performed in this work.

#### Incorrect calculation of factorial stratification analysis

Factorial stratification analyses represent the first step for calculating ORs for stratified data as mentioned in the Introduction, and they have been widely used for stratification analysis in some retrospective studies [9–11]. In these meta-analyses, a simple but incorrect calculation method was employed to combine original data. Stratified data from different studies were simply added together as in a single case-controlled study without considering the heterogeneity between them [9, 11]. This method may cause abnormal results or erroneous conclusions.

**Figure 3:**
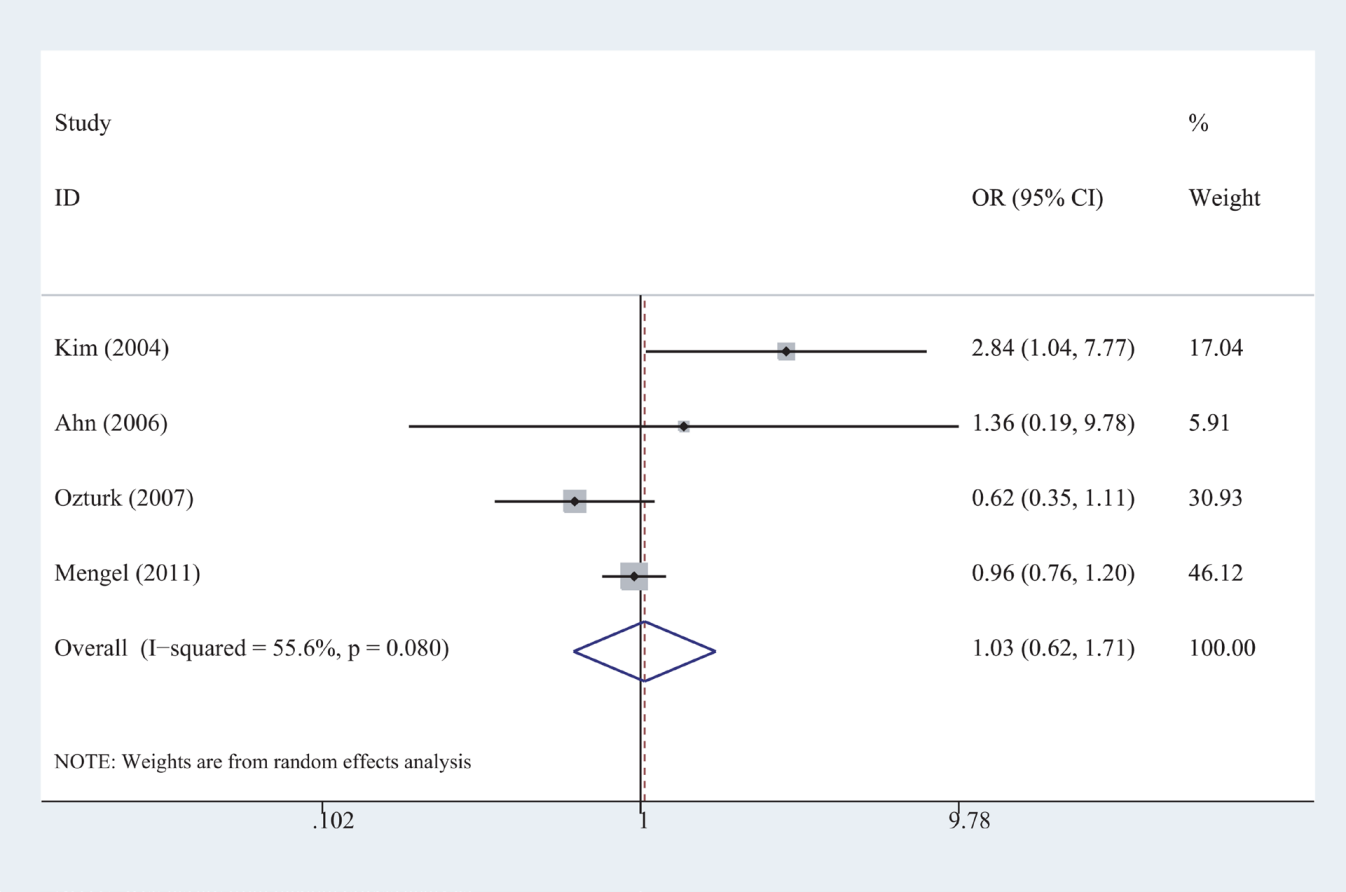
Forest plots of the meta-analysis of the association between AD risk and the CHAT rs3810950 polymorphism among non-ApoE ε4 carriers under the recessive model

In one of our previous studies, we conducted a stratification analysis to investigate the association of the *CHAT* rs3810950 polymorphism with AD risk according to ApoE ɛ4 carrier status [10]. First, we synthesized the original data as a single case-controlled study and obtained an abnormal result in which the CHAT rs3810950 polymorphism played a protective role against the AD risk among non-ApoE ɛ4 carriers (OR = 0.82, *P* = 0.014, shown in Table 5A), which was entirely inconsistent with the results showing the destructive role of this polymorphism obtained from the meta-analysis based on the overall population [10]. After visual inspection of the original data, we found significant variance among the included studies. Therefore, we determined that simply adding study sample numbers together without considering the heterogeneity between studies is incorrect. A random-effects model (the Der Simonian-Laird method) was then used to calculate the effect size. A statistical analysis provided a much better result and showed an OR of 1.03, which indicated no association between the *CHAT* rs3810950 polymorphism and the AD risk among non-ApoE ε4 carriers (OR = 1.03, *P* = 0.08, shown in Table 5B). With the random-effects model, the OR for the combined effect of the *CHAT* rs3810950 polymorphism and ApoE ɛ4 alleles was larger than that for the unilateral effect of ApoE ɛ4 (OR = 4.87, *P* = 0.004 vs. OR = 3.46, *P* = 0.001), which is quite reasonable. However, an opposite trend (OR = 2.07, *P* < 0.001 vs. OR = 4.31, *P* < 0.001) was observed by simple calculation, as in a single case-controlled study calculation. The forest plot from the random-effects model showed that significant between-study heterogeneity existed in the genetic comparison among non-ApoE ε4 carriers (*I*^*2*^ = 55.6%), and two studies with OR values less than 1 had relatively larger weights than the other two, which caused the combined CI to cross 1 (Figure 3). After employing the random-effects model, the weights of these two studies significantly decreased; therefore, the bias in the results also decreased compared with that of the fixed-effect model or by directly adding the samples together as a pseudo single case-controlled study.

**Table 5:** Risk of Alzheimer’s disease associated with the CHAT rs3810950 polymorphism by ApoE 4 status

(A)																
Gene local		Genetic Comparison	non-ApoE 4 carriers						ApoE 4 carriers							
			Cases	Controls	OR		95% CI	*P*	Cases	Controls		OR	95% CI			*P*
*CHAT*	rs3810950	**GG + GA**	851	1605	1 (reference)			NA	862	377		4.31	3.72–4.99			<0.001
		**AA**	292	673	0.82		0.70–0.96	0.014	203	185		2.07	1.67–2.57			<0.001
**(B)**																
**Gene local**		**Genetic Comparison**	**No. of Studies**	**non-APOE 4 carriers**					**APOE 4 carriers**							
				***I***^***2***^	**OR**		**95% CI**	***P***	***I***^***2***^	**OR**					**95% CI**	***P***
*CHAT*	rs3810950	**GG + GA**	4		1 (reference)			NA	94.10%	3.46					1.78–6.71	0.001
		**AA**		55.60%	1.03		0.62–1.71	0.08	77.30%	4.87					1.67–14.22	0.004

Note: OR: odds ratio; CI: confidence interval.

CHAT: Choline acetyltransferase; ApoE ε4: the ε4 allele of the apolipoprotein E gene.

This case suggested that the simple calculation method is likely to cause errors when heterogeneity is high between studies. Combining stratified data using either a fixed-effect or random-effects model provides more stable and accurate results because variation between studies is considered. In the methodology section of this paper, we always used this computing method to calculate the ORs for stratified data.

#### Potential limitations

Although standard stratification analysis has advantages for controlling confounders and revealing potential interactions between exposure and confounding factors, three potential limitations can be recognized in meta-analyses. First, the statistical results of standard stratification analysis may suggest the existence of a potential interaction but cannot quantify its strength. In confounder-controlling stratification analysis (*Step II*), the homogeneity test is performed to estimate variants across strata. Significant variants indicate a potential interacting effect between the stratified moderator and the investigated factor. However, effect sizes of strata are not combined but reported respectively, or further statistical analyses of biological interactions are performed to verify the finding. Second, standard stratification analysis may not be suitable for data with a large number of concomitant variables. With an increasing number of concomitant variables, the number of strata will increase and the actual frequencies of cases or controls within each stratum will decrease, possibly resulting in failure to achieve the necessary conditions of the hypothetical test. Finally, standard stratification analysis cannot be performed when the stratified data are incomplete. In fact, all statistical techniques, including logistic regression methods, require complete data to estimate the difference between the theoretical frequency and the actual frequency (or the expected value and the observed value). Data deficiency always causes difficulty in performing statistical analyses in meta-analyses and systematic reviews; however, researchers can contact the authors of original publications to ask for the missing data. Despite these restrictions, this method can be effectively used by researchers conducting meta-analyses to synthesize data from multiple case-control studies or cohort studies to determine more accurate estimations and more robust conclusions.

With the development of sequencing technology and the increasing number of genome-wide association studies (GWASs) conducted, an increasing number of issues, such as gene-gene or gene-environment effects on disease development, will occur. By unifying the superior aspects of previous stratification methods, a standard stratification analysis will be applicable in controlling for confounding variables and exploring the effects of their interactions on the development of complicated diseases on a multivariate level.

## CONCLUSIONS

This paper systematically introduced the methodology, computing method and application of stratification analyses for meta-analyses. Our main contributions include the (a) establishment of a computing method and interpretation of the results of standard stratification analyses; (b) differentiation between stratification and subgroup analyses; and (c) discussion and resolution of incorrect computing methods for factorial stratification that are frequently used in previous studies. The examples here provide a good understanding of the methodology for standard stratification analyses used for meta-analyses, and they also show the important role of this method in controlling for confounding variables and identifying interacting effects in genetic association studies. More extensive multi-centre studies designed to determine gene-gene, gene-drug and gene-environment interactions are necessary to authenticate this standard stratification method in the future.

## SUPPLEMENTARY MATERIALS TABLES


