# Supplementary material for: Controlling for confounding factors and revealing their interactions in genetic association meta-analyses: a computing method and application for stratification analyses
Source: Oncotarget. 2018 Jan 29;9(15):12125–36. doi: 10.18632/oncotarget.24335 (PMC5844733; doi:10.18632/oncotarget.24335)
Supplement: Supplementary file 1 [file oncotarget-09-12125-s001.pdf]

Controlling for confounding factors and revealing their interactions in genetic association meta-analyses: a computing method and application for stratification analyses

SUPPLEMENTARY MATERIALS

Supplementary Table 1: Stratified data of the three included meta-analysis studies for illustration

Gene local	Assoc allele	First author	Year	Sample size		Strata	Case			Control		
				Case	Control		WW	WM + MM		WW	WM + MM	
PTGS2 rs5275	T>C					NSAID use status						
		Lurie	2010	948	1532	+	172	194		361	344	
						–	282	300		375	452	
		Andersen	2009	359	765	+	53	61		93	144	
						–	94	151		222	306	
		Barry	2009	427	536	+	118	156		163	200	
						–	72	81		70	103	
		Gong	2009	162	211	+	14	14		15	46	
						–	50	84		54	96	
		Vogel	2008	399	737	+	54	69		90	139	
						–	125	151		218	290	
		Vogel	2007	301	312	+	29	49		46	49	
						–	92	131		97	120	
		Vogel	2006	356	356	+	92	108		103	119	
						–	73	83		50	84	
		Gallicchio	2006	80	1263	+	9	5		158	198	
						–	29	37		396	511	
CYP46A1 rs754203	T>C					ApoE ε4 status	WW	WM	MM	WW	WM	MM
		Desai (1)	2002	434	398	+	125	115	28	33	34	12
						–	90	59	17	158	132	29
		Desai (2)	2002	54	61	+	23	9	1	10	4	1
						–	13	8	0	32	13	1
		Combarros	2004	321	315	+	98	64	15	30	23	2
						–	79	53	12	161	91	8
		Ingelsson	2004	173	91	+	58	42	8	14	9	0
						–	30	26	9	28	37	3
		Wang	2004	99	113	+	25	5	0	5	7	1
						–	52	15	2	56	42	2
		Golanska	2005	215	173	+	53	56	20	30	13	1
						–	36	38	12	55	62	12
		Li	2006	108	105	+	23	17	2	12	8	0
						–	23	37	6	49	29	7
		Tedde	2006	296	177	+	72	63	9	9	6	0
						–	75	68	9	82	72	8
MTHFR rs1801133	C>T	Chapman	1998	49	34	+	4	17	1	9	11	2
						–	8	14	5	5	5	2
		Brunelli	2001	231	137	+	28	54	19	3	4	5
						–	36	66	28	36	61	28
		Religa	2003	99	100	+	30	22	4	13	6	2
						–	23	16	4	42	32	5
		KIDA	2004	194	379	+	38	52	12	25	30	5
						–	26	46	20	119	163	37
		Wang	2005	104	130	+	12	12	4	12	4	0
						–	38	26	12	67	43	4
		Bi	2008	386	375	+	52	98	75	17	28	10
						–	30	81	50	73	144	103

Supplementary Table 2: Meta-analysis for the associations of the PTGS2 rs5275 polymorphism and NSAID use with the risk of cancer

Meta-analysis	No. of studies	Genetic comparison	Non-NSAID user					NSAID user				
			I^2	Model	OR	95% CI	*P*	I^2	Model	OR	95% CI	*P*
Nagao M et al., 2013	8	**TT**	NA		1 (reference)		NA	NA		1 (reference)		NA
		**TC + CC**	0.00%	F	0.934	0.828–1.053	0.264	49.80%	F	1.008	0.872–1.165	0.916
			**TT**					**TC + CC**				
		**Non-NSAID user**	NA		1 (reference)		NA	NA		1 (reference)		NA
		**NSAID user**	48.60%	F	0.769	0.665–0.889	<0.001	40.20%	F	0.841	0.738–0.959	0.01

Note: OR: odds ratio; CI: confidence interval; F: fixed-effect model; NA: not available; PTGS2: prostaglandin endoperoxide synthase 2; NSAID: nonsteroidal anti-inflammatory drug.

Supplementary Table 3: Meta-analysis for the associations of the CYP46A1 rs754203 or MTHFR rs1801133 polymorphisms with the risk of Alzheimer's disease by ApoE ε4 status

Meta-analysis	Gene local	No. of studies	Genetic comparison	Non-ApoE ε4 carrier					ApoE ε4 carrier				
				I^2	Model	OR	95% CI	*P*	I^2	Model	OR	95% CI	*P*
Li L et al., 2013	*CYP46A1* rs754203	8	**TT + TC**	NA		1 (reference)		NA	NA		1 (reference)		NA
			**CC**	0.00%	F	1.528	1.075–2.172	0.018	33.50%	F	1.33	0.790–2.239	0.284
Zhang M et al., 2010	*MTHFR* rs1801133	6	**CC**	NA		1 (reference)		NA	NA		1 (reference)		NA
			**TT**	39.50%	F	1.557	1.119–2.165	0.009	12.70%*	F*	1.619*	0.935–2.805*	0.086*

Note: OR: odds ratio; CI: confidence interval; F: fixed-effect model; R: random-effect model; NA: not available; CYP46A1: cholesterol-24S-hydroxylase; ApoE ε4: the ε4 allele of the apolipoprotein E gene; MTHFR: methylenetetrahydrofolate reductase; *Calculated by us for illustration.

Code of command line in Stata

****Step I: factorial stratification analysis**

** Defining available variable
gen c = caseNonexposure0
gen d = conNonexposure0
gen a1 = caseNonexposure1
gen b1 = conNonexposure1
gen a2 = caseExposure0
gen b2 = conExposure0
gen a3 = caseExposure1
gen b3 = conExposure1

**Calculating the pooled OR with a 95% CI to reveal the effect of the stratifying indicator within the unexposed group (OR_{2-}) using the fixed-effect model
metan a1 b1 c d, label(namevar = study, yearvar = year) fixed or
**Calculating the pooled OR with a 95% CI to reveal the effect of the stratifying indicator within the unexposed group (OR_{2-}) using the random-effect model
metan a1 b1 c d, label(namevar = study, yearvar = year) random or

**Calculating the pooled OR with 95% CI to reveal the effect of the exposure factor within the same stratum (OR_{1+}) using the fixed-effect model
metan a2 b2 c d, label(namevar = study, yearvar = year) fixed or
**Calculating the pooled OR with 95% CI to reveal the effect of the exposure factor within the same stratum (OR_{1+}) using the random-effect model
metan a2 b2 c d, label(namevar = study, yearvar = year) random or-

**Calculating the pooled OR with 95% CI to reveal the combined effect of both the stratifying indicator and the exposure factor (OR_{2+}) using the fixed-effect model
metan a3 b3 c d, label(namevar = study, yearvar = year) fixed or
**Calculating the pooled OR with 95% CI to reveal the combined effect of both the stratifying indicator and the exposure factor (OR_{2+}) using the random-effect model
metan a3 b3 c d, label(namevar = study, yearvar = year) random or

****Step II: confounder-controlling stratification analysis**

**Calculating the pooled OR with 95% CI to reveal the effect of the exposure factor within stratum 1 (OR_1) using the fixed-effect model
metan a2 b2 c d, label(namevar = study, yearvar = year) fixed or
**Calculating the pooled OR with 95% CI to reveal the effect of the exposure factor within stratum 1 (OR_1) using the random-effect model
metan a2 b2 c d, label(namevar = study, yearvar = year) random or

**Calculating the pooled OR with 95% CI to reveal the effect of the exposure factor within stratum 2 (OR_2) using the fixed-effect model
metan a3 b3 a1 b1, label(namevar = study, yearvar = year) fixed or
**Calculating the pooled OR with 95% CI to reveal the effect of the exposure factor within stratum 2 (OR_2) using the random-effect model
metan a3 b3 a1 b1, label(namevar = study, yearvar = year) random or

**Overall estimation for the effect of the investigated factor by combing the ORs from each stratum (Combined OR)
gen logor = log(or)
gen loglci = log(lci)
gen loguci = log(uci)
metan logor loglci loguci, label(namevar = stratum) fixed effect(OR) eform
